# Dose-escalated radiotherapy with PET/CT based treatment planning in combination with induction and concurrent chemotherapy in locally advanced (uT3/T4) squamous cell cancer of the esophagus: mature results of a phase I/II trial

**DOI:** 10.1186/s13014-021-01788-4

**Published:** 2021-03-23

**Authors:** C. Pöttgen, E. Gkika, M. Stahl, J. Abu Jawad, T. Gauler, S. Kasper, T. Trarbach, K. Herrmann, N. Lehmann, K.-H. Jöckel, H. Lax, M. Stuschke

**Affiliations:** 1 Department of Radiation Oncology, West German Cancer Centre, University of Duisburg-Essen, Hufelandstr. 55, 45147 Essen, Germany; 2 Department of Radiation Oncology, University Hospitals Freiburg, Freiburg, Germany; 3 Department of Medical Oncology and Hematology, Evang. Kliniken Essen-Mitte, Essen, Germany; 4 Department of Medical Oncology, West German Cancer Centre, University of Duisburg-Essen, Essen, Germany; 5 Center for Tumor Biology and Integrative Medicine, Klinikum Wilhelmshaven, Wilhelmshaven, Germany; 6 Department of Nuclear Medicine, West German Cancer Centre, University of Duisburg-Essen, Essen, Germany; 7 Institute of Medical Informatics, Biometry and Epidemiology, University of Duisburg-Essen, Essen, Germany

**Keywords:** Esophageal cancer, Squamous cell carcinoma, Dose-escalation, Definitive chemoradiation

## Abstract

**Background:**

This prospective phase I/II trial assessed feasibility and efficacy of dose-escalated definitive chemoradiation after induction chemotherapy in locally advanced esophageal cancer. Primary study endpoint was loco-regional progression-free survival at 1 year.

**Methods:**

Eligible patients received 2 cycles of induction chemotherapy with irinotecan, folinic acid and 5-fluorouracil weekly and cisplatin every 2 weeks (weeks 1–6, 8–13) followed by concurrent chemoradiation with cisplatin and irinotecan (weeks 14, 15, 17, 18, 20). Radiotherapy dose escalation was performed in three steps (60 Gy, 66 Gy, 72 Gy) using conventional fractionation, planning target volumes were delineated with the aid of 18F-FDG-PET/CT scans. During follow-up, endoscopic examinations were performed at regular intervals.

**Results:**

Between 09/2006 and 02/2010, 17 patients were enrolled (male/female:13/4, median age: 59 [range 48–66] years, stage uT3N0/T3N1/T4N1: 4/12/1). One patient progressed during induction chemotherapy and underwent surgery. Of 16 patients treated with definitive chemoradiotherapy, 9 (56%) achieved complete response after completion of chemoradiation. One-, 2-, 3- and 5-year overall survival rates (OS) were 77% [95%CI: 59–100], 53% [34–83], 41% [23–73], and 29% [14–61], respectively. Loco-regional progression-free survival at 1, 3, and 5 years was 59% [40–88], 35% [19–67], and 29% [14–61], corresponding cumulative incidences of loco-regional progressions were 18% [4–39%], 35% [14–58%], and 41% [17–64%]. No treatment related deaths occurred. Grade 3 toxicities during induction therapy were: neutropenia (41%), diarrhoea (41%), during combined treatment: neutropenia (62%) and thrombocytopenia (25%).

**Conclusions:**

Dose-escalated radiotherapy and concurrent cisplatin/irinotecan after cisplatin/irinotecan/5FU induction chemotherapy was tolerable. The hypothesized phase II one-year loco-regional progression free survival rate of 74% was not achieved. Long-term survival compares well with other studies on definitive radiotherapy using irinotecan and cisplatin but is not better than recent trials using conventionally fractionated radiotherapy ad 50 Gy with concurrent paclitaxel or 5FU and platinum compound.

*Trial registration* The present trial was registered as a phase I/II trial at the EudraCT database: Nr. 2005-006097-10 (https://www.clinicaltrialsregister.eu/ctr-search/trial/2005-006097-10/DE) and authorized to proceed on 2006-09-25.

## Introduction

Esophageal cancer has become the seventh most common cancer worldwide but prognosis remains poor (WHO 2018) [1]. Patients with locally advanced tumors (T3–T4 N0-1 M0) are a domain of combined preoperative or definitive chemoradiotherapy but experienced long-term survival rates of about only 14–26% [2, 3].

Long-term survival rates at 3 years after definitive radiochemotherapy at a total dose of 50.4 Gy approach about 20–50% in recent prospective trials [4–6]. In the past, randomized studies have demonstrated promising results of combined definitive radiochemotherapy not inferior to multimodality protocols including surgery especially in responders after induction chemotherapy [7, 8]. Loco-regional recurrences remain the dominant risk for the patient after definitive radiochemotherapy [4–6, 9].

Patients who show clinical response to induction treatment (chemo- or chemo-radiotherapy) have the best prognosis and according to the data of the German Esophageal Cancer Study Group long-term survival of about 50% can be achieved in this group of patients with definitive radiochemotherapy [7]. Overall, local (in-field) disease recurrences remain a major therapeutic problem after radiochemotherapy and account for 65% of all disease relapses.

Consequently, improving induction chemotherapy to increase the proportion of “responders”, as well as intensification of the radiotherapy by dose-escalation could be a strategy to improve local disease control.

Recent benchmark randomized trials showed that combined radiochemotherapy was more effective than radiotherapy alone, but radiation dose escalation during concurrent chemotherapy did not result in a better survival [9–11]. The comparison of randomized trials using surgery after neoadjuvant chemoradiotherapy and those with definitive chemoradiotherapy did not show significant differences regarding survival despite using maximum local treatment, e.g. radical resection [12]. The benefit of radiation dose-escalation may depend on the type of concurrent chemotherapy. Here, we have conducted a prospective trial on the basis of induction chemotherapy with irinotecan added to cisplatin/5-FU/FA and a stepwise dose-escalation of three-dimensional conformal radiotherapy with PET/CT based treatment planning. The experience of a good tolerance to combined chemoradiotherapy schedules with cisplatin/irinotecan and promising rates of pathologic remissions form the basis for this investigation [13–15]. A more recent randomized trial comparing neoadjuvant radiochemotherapy with cisplatin/irinotecan with carbo/paclitaxel found similar overall survival, pCR rate and tolerability [16].

## Patients and methods

This is an oligocentric prospective phase I/II trial (EudraCT-Nr. 2005-006097-10). Patients were accrued from November 2006 to February 2010. The trial was approved by the Ethics Committee of the University of Duisburg-Essen as well as by the national legal authorities. All patients provided written informed consent. Primary study endpoint was loco-regional progression-free survival at one year. Secondary endpoints were: rate of objective remissions, treatment lethality, overall survival at 2 years, distant metastasis rates, and rates of acute and chronic toxicities.

### Eligibility

Patients of 18–70 years with biopsy-proven squamous cell carcinoma of the esophagus, up to 3 cm above the anatomic cardia, with locally advanced disease (uT3–4 anyN M0), according to the American Joint Committee of Cancer (6th Edition, 2003) were eligible.

In addition, good clinical condition (WHO performance status 0 to 1) with normal liver (bilirubin < 1.5 mg/dl, cholinesterase > 3000 U/l, total protein > 60 g/l), renal (creatinine clearance > 60 ml/min, creatinin < 1,3 mg/dl) and bone marrow function (leukocytes > 4000/μl, thrombocytes > 150,000/μl, Hb > 10 g/dl) were prerequisites for study enrolment. Patients should be fit for surgery (‘medical operability’: no cardio-pulmonary insufficiency, ejection fraction > 2.5 l/min, arterial pO2 > 65 mmHg, FEV1 > 70%).

This study was planned as a phase I/II study for optimization of definitive radiochemotherapy based on the results of arm B of our previous phase III study. Consequently, surgery was not planned within the study, and offered only after study dropout (e.g. in case of minor response and/or inadequate symptom relief). An upfront interdisciplinary decision that definitive radiochemotherapy represents the preferable treatment option and surgery is not offered in the primary treatment after neoadjuvant radiochemotherapy was presumed.

### Pretreatment evaluation

All patients underwent pretreatment staging including physical examination, cardiopulmonary function tests, routine hematologic and biochemical tests, esophageal barium swallows, upper gastrointestinal endoscopy with histological biopsy, EUS and PET/CT. After completion of the induction chemotherapy patients again underwent barium swallow and endoscopy. A second PET/CT scan was planned at this time point for radiotherapy treatment planning.

### Treatment

#### Chemotherapy

**Fig. 1:**
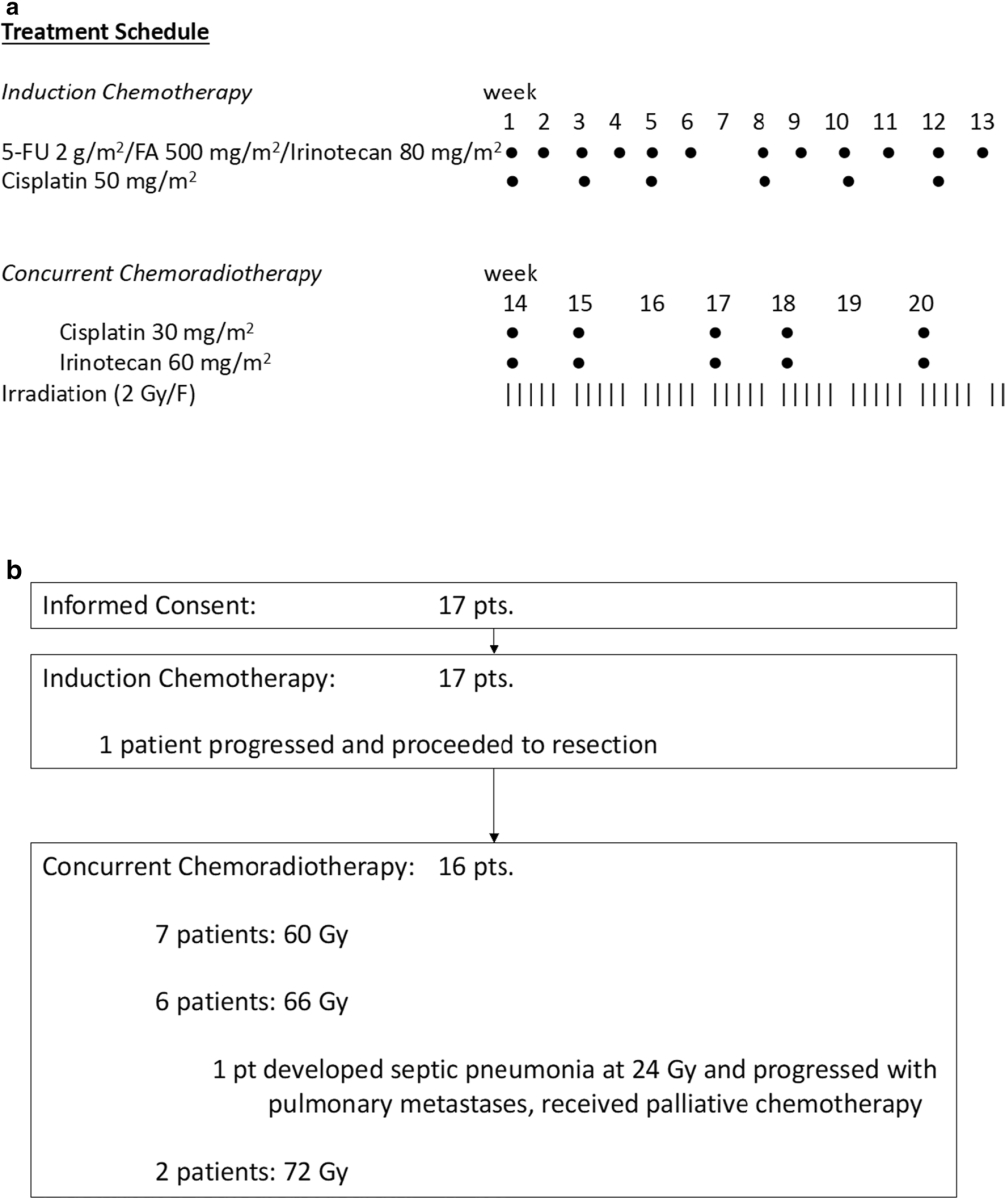
**a** Study scheme. **b** CONSORT diagram

Patients received 2 cycles of induction chemotherapy with cisplatin 50 mg/m^2^ (1 h infusion) on weeks 1,3,5 and 8,10,12 combined with 5-FU 2 g/m^2^ (24 h-infusion), folinic acid (FA) 500 mg/m^2^ (2 h infusion) and irinotecan 80 mg/m^2^ (1 h infusion, weekly during weeks 1–6 and 8–13). This regimen was followed by concurrent chemoradiation with cisplatin 30 mg/m^2^ and irinotecan (Iri) 60 mg/m^2^ on day 1, 8, 22, 29, 43 (Fig. 1). Adequate hydration, antiemetics, and supportive medications were administered. Non-hematologic toxicities > grade 1, according to the common terminology criteria of adverse events v3.0 (CTCAE), lead to treatment delay of one week and > grade 2 to an additional reduction of 5-FU to 1.6 g/m^2^. Neutro- and thrombocytopenias > grade 1 on treatment day resulted in discontinuation of irinotecan, and neutropenia > grade 3 as well as thrombocytopenia of > grade 2 resulted in a delay of chemotherapy and a dose reduction of cisplatin and irinotecan for upcoming therapies. Toxic nephro- and neuropathy > grade 1 resulted in discontinuation of cisplatin. If there was a disease progression after induction chemotherapy patients were excluded from the study and were offered individual treatment.

#### Radiotherapy

The macroscopic tumor volume (GTV) was defined using all information from the findings of initial EUS and PET/CT before and after induction therapy. The initial clinical target volume (CTV1) was constructed from the pretreatment GTV with an axial margin of 0.5 to 1 cm and craniocaudal (cc) margins of 4 cm with respect to anatomic boundaries. For the planning target volume (PTV1) the CTV1 was extended circumferentially with margins of 0.5 to 1 cm. PTV1 was planned to receive 50 Gy in 25 fractions.

For the boost volume the GTV was delineated according to the pre- and post-induction chemotherapy PET/CTs and CTs (GTV1: gross tumor volume based on pre-induction scans, GTV2: gross tumor volume based on post-induction scans). The CTV2 included the GTV2 with an axial margin of 0.5 to 1 cm and a cranio-caudal margin of 1 cm; the PTV2 included the CTV2 with a 0.5–0.7 cm axial and a 1 cm cranio-caudal margin. For PTV2, a risk adapted dose escalation was intended. In the first escalation level (level 1) the PTV1 received a dose of 60 Gy, in the second escalation level (level 2) a total dose of 66 Gy, and in the third escalation level (level 3) the PTV2 received 72 Gy. Predefined dose limiting toxities were: non-malignant ulcerations, treatment-related death, tumor associated bleedings.

The escalation schedule depended on the incidence of dose limiting toxicities. If there were no ulcerations described in the first 5 patients within 2 months after therapy completion, the next 5 patients proceeded to the next escalation level. At the presence of up to 2 treatment related ulcerations, the next 5 patients did not proceed to the next escalation level.

The maximum spinal cord dose permitted was 42 Gy, the mean lung dose was restricted to 17 Gy and the total lung volume receiving > 20 Gy (V_20_) was to be kept below 30%, the total myocardium volume receiving > 45 Gy was < 65%. The volume of the esophagus receiving more than 50 Gy should not exceed 12 cm length.

### Follow-up evaluation and toxicity criteria

After treatment completion, patients were evaluated every three months for the first two years, then every 6 months for the next three years, then annually including physical examination, cardiopulmonary function tests; routine hematologic and biochemical tests; EUS with biopsy of suspect lesions and CTs of thorax and abdomen. Toxicity was documented according to the common terminology criteria of adverse events v3.0 (CTCAE).

### Statistical analysis

The Kaplan Meier method was used to estimate overall survival (OS), progression-free survival (PFS) and loco-regional control (LRC) probability. For the latter, death and loss of follow-up were considered as censoring events. The log-rank test was used for univariate analysis of prognostic factors and Cox proportional hazard model was used for multivariate analysis. A significance level of *p* = 0.05 was used, all tests were two-sided. For phase I radiotherapy dose escalation, the following phase I rule was applied:

Starting at a total dose of 60 Gy, 5 patients are treated at the respective dose level, if nill therapy related and not tumor-associated esophageal ulcers of Grade III-V occur in the treated volume then proceed to the next dose level. If 1–2 ulcers occur, then treat another 5 patients. If there are ≤ 2 ulcers among the 10 treated patients in this dose group than proceed to the next dose level. Otherwise, continue the trial at the previous dose level, if one is at an escalated dose level or stop the trial, if one is at the base total dose level. The study would have been stopped if more than 3 therapy-related deaths would have occurred among the first 20 patients. Primary end-point for the phase II part of this trial: Under H0, loco-regional progression free survival (loco-regional PFS) was assumed to be 54% at 1 year follow-up as in our previous trial on resectable locally advanced squamous cell carcinomas of the esophagus [7], under H1, loco-regional PFS was expected to be ≥ 74%. In addition, the prognostic value of the gross tumor volume (GTV), total radiation dose, and PET-response to induction chemotherapy was analysed.

Per protocol, no cut-off values had been pre-specified for classification of patients as metabolic responders using pre- and post-induction PET scans. Thus, during the present analysis, %SUV remaining was taken as a continuous parameter in multivariable analysis and having a positive finding, a median split was chosen as threshold value for visualisation of the influence. Patients who had lower SUV values than the corresponding median (response to induction chemotherapy: responder vs. non-responder measured as deltaSUV_max_ = SUV_max_(after Induction)/SUV_max_(before Induction): ≤ median vs. > median) were classified as responders, the remaining patients as non-responders, respectively.

In addition, for the following factors cut-off levels were used: initial GTV volume ≤ median versus > median; radiation dose: ≤ 60 Gy versus > 60 Gy. Statistical analysis was performed using SAS (Version 9.4, SAS Institute, Cary, NC) and R (version 3.6.1, R Core Team (2019)) [17, 18].

Cumulative incidences based on competing risk analysis were calculated with R package ‘cmprsk’ [19].

## Results

### Patients

Seventeen patients were enrolled from September 2006 until February 2010. The patient and treatment characteristics are listed in Table 1.

**Table 1:** Patient and treatment characteristics (of the patients undergoing combined chemoradiotherapy)

Characteristics	No. (%)
Age (median: 59, range: 46–65 years)	
< 60 years	9 (53)
≥ 60 years	8 (47)
Sex	
Male	13 (77)
Female	4 (23)
Clinical T stage	
T3 N0	4 (24)
T3 N1	12 (70)
T4 N1	1 (6)
Pretreatment metabolic tumor volume	
Median 33.2 ml (range: 4.5–195.3 ml)	
Pretreatment SUV_max_	
Median 13.7 (range: 2.8–20.9)	
Postinduction SUV_max_	
Median 2.95 (range: 2.2–12.4)	
Metabolic response (SUV_max[post-induction]_/SUV_max[pretreatment]_)	
Median 0.39 (range: 0.1–1.3)	
Response to ICT	
Responders	12 (71)
Non-responders	5 (29)
Concurrent chemoradiation	
Complete response	9 (56)
Evidence of disease	7 (44)
Physical dose	
< 66 Gy	9 (56)
≥ 66 Gy	7 (44)

All patients had T3-/T4-tumors proven by endosonography and were discussed interdisciplinary. Most had upper third carcinomas. Surgery was not offered due to high tumor localization, extensive nodal involvement, and/or patient’s denial.

One patient showed local progression during induction chemotherapy, underwent surgery and was resected. The remaining patients proceeded to concurrent radiochemotherapy (Fig. 1b). The trial was closed after more than 3 years of recruitment due to slow accrual.

### Induction chemotherapy

All patients received two cycles of induction chemotherapy according to the protocol except one who changed to another drug combination (cisplatin/5FU/FA) due to subject-related complaints. During induction chemotherapy 7 patients (41%) developed grade 3 neutropenia, one patient grade 3 and one patient grade 4 thrombocytopenia. A grade 3 diarrhoea occurred in 7 (41%) patients. One patient experienced bradycardia after the first course of chemotherapy which needed subsequent pacemaker implantation, one patient suffered stenocardia, and one patient developed an acute femoral occlusion requiring acute intervention.

Objective responses after induction chemotherapy were observed in 12 of 17 patients (71%, 2 complete remissions, 10 partial remissions). One patient was evaluated as progressive, was taken from study, underwent surgery and was resected, further 4 patients showed stable disease. Apart from the patient who progressed during induction chemotherapy, no further patients underwent subsequent surgery. The remaining patients proceeded to concurrent radiochemotherapy (Fig. 1b).

Metabolic responses with SUV_max_ decreases below 35% of the initial SUV_max_ were observed in 6 patients (see Table 1), the median deltaSUV was 39%.

### Chemoradiation

Overall, 7 patients were assigned to 60 Gy total dose and received 60 Gy, 7 patients were assigned to 66 Gy and 6 patients received 64–66 Gy (1 patient rejected the last fraction). One patient planned for dose escalation level 2 (66 Gy) actually received 24 Gy until development of septic candida pneumonia with pulmonary insufficiency which resulted in a treatment break and disease progression with pulmonary metastases in the subsequent CT scans. Two patients were irradiated up to a total dose of 72 Gy. The study was closed early due to slow accrual.

During chemoradiation 10 patients developed grade 3 neutropenia (63%), grade 3 thrombocytopenia was observed in 3 patients (19%), grade 4 thrombocytopenia in 2 patients (13%). Five patients received concurrent chemotherapy applications as planned while in all other patients treatment reductions or modifications were necessary. One patient received concurrent chemotherapy according to the RTOG 85-01 protocol (cisplatin/5-FU) due to the side-effects of induction chemotherapy.

Non-hematological grade 3 toxicities were found during radiochemotherapy in one third of the patients, esophagitis greater than grade 2 was found in 2 patients. Grade 4 toxicities or treatment related deaths were not observed.

### Efficacy

Two months after chemoradiation 7 patients had biopsy proven residual disease and 9 (53%) had a complete remission.

Two patients showed early local progression after definitive chemoradiotherapy. Salvage surgery was discussed but was not performed due to tracheo-bronchial infiltration in one patient, and patient denial of resection in the other.

Loco-regional progression inside the high dose volume was observed in 9 patients overall: 6 patients in the group receiving 60 Gy, and 3 patients in the group receiving > 60 Gy. All progression sites were concordant with FDG-avid areas after induction.

Out-field recurrences, mainly in adjacent lymph node areas, were detected in 3 patients.

Four of the patients in complete remission developed a local recurrence. Metastases occurred in 6 patients (4 pulmonary, 2 hepatic).

Two patients developed head and neck tumors as second malignancy (location: nasopharyngeal, base of tongue).

During follow-up (median 147 months) 15 patients died, 14 due to disease progression, or metastasizing second malignancy (one patient), respectively.

**Fig. 2:**
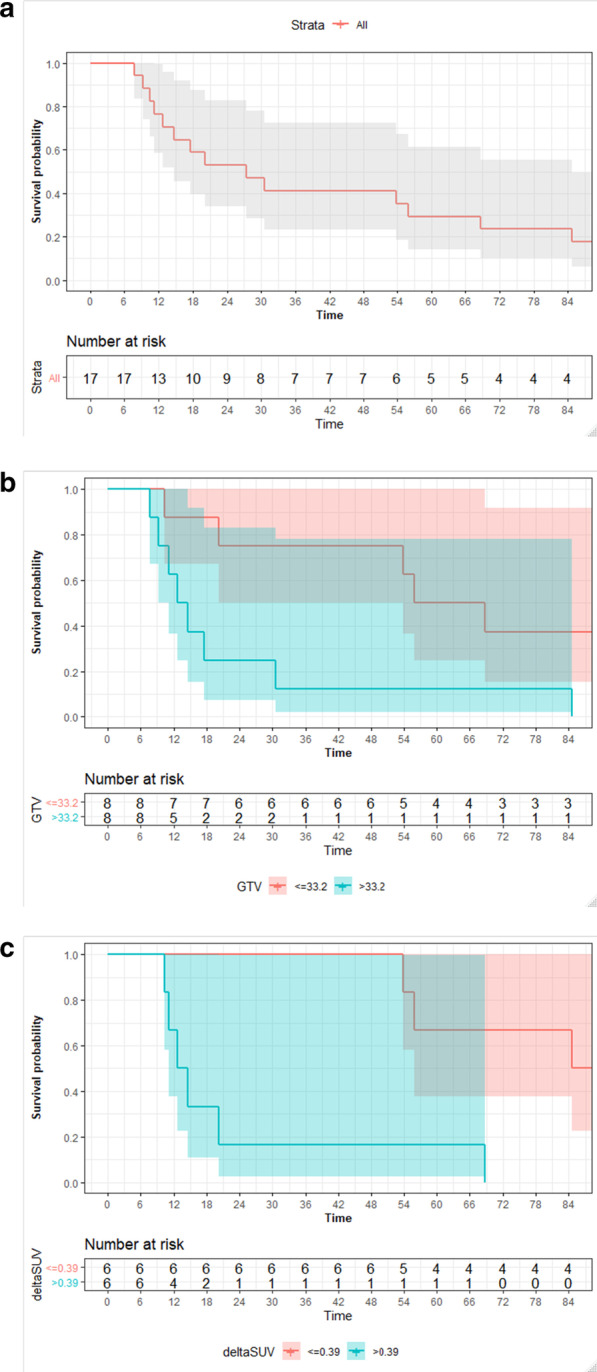
**a** Overall survival of all patients (n = 17). **b** Overall survival of all patients treated with concurrent radiochemotherapy (n = 16), stratified by median pretreatment gross tumor volume (≤ 33.2 ml vs > 33.2 ml). **c** Overall survival of all patients treated with concurrent radiochemotherapy (n = 16), stratified by median metabolic response to induction chemotherapy (deltaSUV = SUV_max[postinduction]_/SUV_max[pretreatment]_ ≤ 0.39 vs deltaSUV > 0.39)

One-, 2-, 3- and 5-year overall survival rates (OS) were 77% [95%CI: 59–100], 53% [34–83], 41% [23–73], and 29% [14–61], respectively (Fig. 2a). Pretreatment gross tumor volume (GTV) was significantly related to overall survival (Fig. 2b). Patients with tumors larger than the median volume of 33.2 ml had 1-, 2-, 3- and 5-year survival rates of 63% [95%CI: 37–100%], 25% [95%CI: 8–83%], 13% [95%CI: 2–78%], and 13% [95%CI: 2–78%], while patients with smaller tumors had corresponding survival rates of 88% [95%CI: 67–100%], 75% [95%CI: 50–100%], 75% [95%CI: 50–100%], and 50% [95%CI: 25–100%], respectively (*p* = 0.02, logrank).

Patients with metabolic response to induction chemotherapy (deltaSUV ≤ 0.39) had significantly better overall survival than patients with less SUV reduction after induction (Fig. 2c): 1-, 2-, 3-, and 5-year survival rates were 100%, 100%, 100%, and 67% [95%CI: 38–100%) in contrast to 67% [95%CI: 38–100%], 17% [95%CI: 3–99%], 17% [95%CI: 3–99%], and 17% [95%CI: 3–99%] (*p* = 0.005, logrank).

Multivariable Cox proportional hazard analysis using several prognostic factors (including age, T-, N-status, pretreatment tumor volume, metabolic response, dose) confirmed metabolic response as single significant effect for overall survival (*p* = 0.03, HR 5.87, 95%CI: 1.08–31.9).

**Fig. 3:**
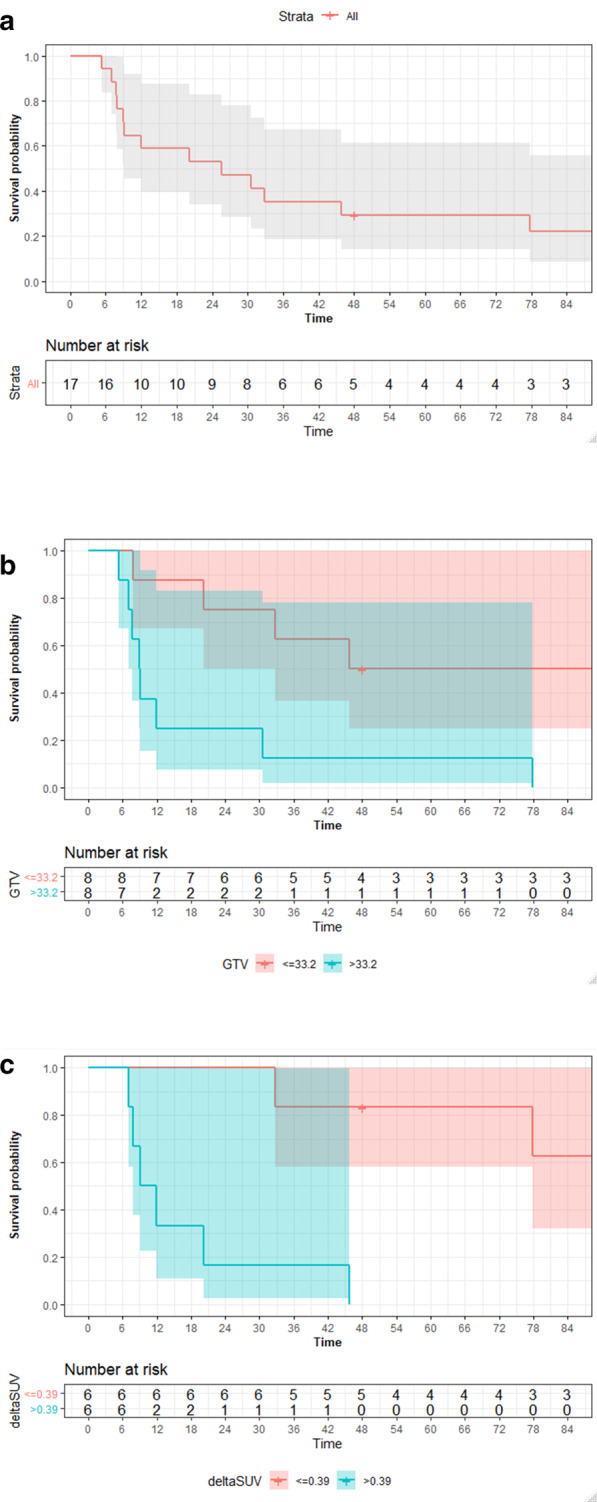
**a** Loco-regional progression-free survival of all patients (n = 17). **b** Loco-regional progression-free survival of all patients treated with concurrent radiochemotherapy (n = 16), stratified by median pretreatment gross tumor volume (≤ 33.2 ml vs > 33.2 ml). **c** Loco-regional progression-free survival of all patients treated with concurrent radiochemotherapy (n = 16), stratified by median metabolic response to induction chemotherapy (deltaSUV ≤ 0.39 vs deltaSUV > 0.39)

**Fig. 4:**
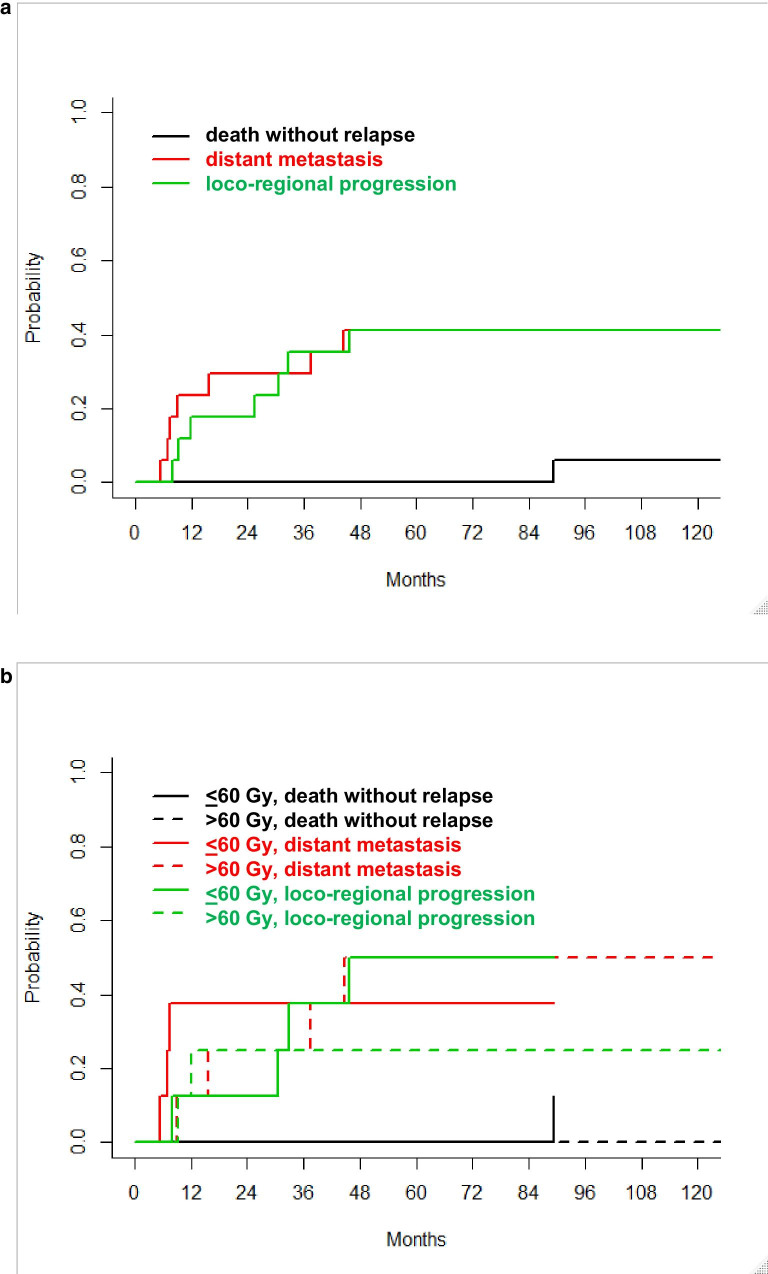
Competing risk analysis. **a** Cumulative incidences of the events: death without relapse (black), distant metastases (red), or loco-regional recurrence (green), all patients (n = 17). **b** cumulative incidences of death without relapse (black), distant metastases (red), or loco-regional recurrence (green), grouped by radiotherapy dose (≤ 60 Gy [solid lines] versus > 60 Gy [dashed lines])

The primary end-point of this study, loco-regional progression-free survival at 1 year was 59% [95%CI: 40–88%] not clinical meaningfully better than the 54% expected under H0 and not approaching the predefined benchmark under H1 of 74%. In addition, loco-regional progression free survival at 3, and 5 years for patients after concurrent radiochemotherapy was 35% [19–67%], and 29% [14–61%] (Fig. 3a). Corresponding cumulative incidences of loco-regional progressions were 18% [4–39%], 35% [14–58%], and 41% [17–64%] (Fig. 4a).

Pretreatment gross tumor volume (GTV) was significantly correlated with better loco-regional progression-free survival rates. Patients with tumor volumes at baseline smaller than 33.2 ml had loco-regional progression-free survival rates at 1, 3, and 5 years of 88% [95%CI: 67–100%], 63% [95%CI: 37–100%], and 50% [95%CI: 25–100%] versus 25% [95%CI: 8–83%], 13% [95%CI: 2–78%], and 13% [95%CI: 2–78%] (*p* = 0.009, logrank, Fig. 3b). Metabolic responder had loco-regional progression-free survival rates at 1, 3, and 5 years of 100%, 83% [95%CI: 58–100%], and 83% [95%CI: 58–100%] versus 33% [95%CI: 11–100%], 17% [95%CI: 3–99%], 17% [95%CI: 3–99%], and 0% (*p* = 0.002, logrank, Fig. 3c).

Metabolic response was confirmed as single significant effect for loco-regional progression-free survival in Cox proportional hazard analysis (HR 11.7, 95%CI: 1.3–106, *p* = 0.028).

Two patients developed esophago-tracheal fistulae related to tumor recurrence and one of them needed tracheotomy two months after chemoradiation due to obstructive larynx edema.

Radiotherapy dose (≤ 60 Gy vs > 60 Gy) was not found to influence loco-regional control. Loco-regional progression-free survival in patients receiving concurrent chemoradiotherapy with doses ≤ 60 Gy was 50% [95%CI: 25–100%], 25% [8–83%], and 13% [2–78%] at 1, 3, and 5 years. Patients in the higher dose group (> 60 Gy) experienced loco-regional progression-free survival rates of 63% [95%CI: 37–100%], 50% [25–100%], and 50% [25–100%] (*p* = 0.2, logrank).

Cumulative incidences of loco-regional relapse in competing risk analysis (death without relapse versus distant metastasis versus loco-regional progression as first event of relapse) were not different between low- and high-dose radiotherapy group (Fig. 4b). Loco-regional progression-rates were 13% [95%CI: 0–46%], 38% [6–71%], and 50% [10–81%] at 1, 3, and 5 years in the group of patients (n = 8) receiving ≤ 60 Gy. The corresponding loco-regional recurrence rates in the group of patients receiving > 60 Gy (n = 8) were 25% [95%CI: 3–58%] at all time points (*p* = 0.4, Fine and Gray’s test, Fig. 4b).

## Discussion

At the time when this study was designed, a series of prospective trials tested induction chemotherapy with cisplatin and irinotecan followed by concurrent chemoradiation with radiotherapy doses at about 50 Gy with or without surgery.

Median survival ranged from 25 to 31 months for the patients treated with trimodality therapy [13, 20–22]. Patients treated with definitive radiochemotherapy had overall survival rates at 2 years ranging from 28 to 42% [14, 23]. In two studies patients received additional cetuximab [22, 23].

Three-year overall survival in the present study was 41%, well in the range of other studies on definitive radiochemotherapy (50 Gy with second- or third-generation chemotherapy) which observed 27–47% [4–6, 11].

Loco-regional progression rates were 35% in this study, well in comparison with 49% in the standard arm of the RTOG 0436 trial [6], and 52% in the standard arm of ARTDECO, respectively [11]. Similarly to the latter study, no significant difference in the local efficacy between radiotherapy dose groups of ≤ 60 Gy versus > 60 Gy could be determined.

Retro- and prospective trials on definitive radiochemotherapy with concurrent cisplatin/irinotecan are rather limited. In relation to the single institution retrospective comparison of Ruppert et al. [24] who found a three-year overall survival rate of 20% (10–41%), and freedom from loco-regional progression of 28% at three years, the present data are more favorable. This applies as well for the comparison with 2-year-overall survival of 28% and 33%, and 24% 2-year-progression-free survival of prospective trials [14, 23].

The incidence of hematologic and non-hematologic toxicities was moderate in the present study similar to other trials [13, 14, 21–23].

A subsequent retrospective trial has claimed a benefit of paclitaxel over irinotecan [24] but a prospective randomized trial on neoadjuvant radiochemotherapy using cisplatin/irinotecan or carboplatin/paclitaxel showed similar results [16].

For squamous cell carcinomas the effect of adding surgery to chemoradiation has been evaluated in randomised trials showing no difference in overall survival for responders to induction chemotherapy although there was a substantial improvement in local control for the surgical arms [7, 8]. A meta-analysis of definitive radiotherapy versus surgery within multimodality protocols confirmed that overall survival was equivalent in both arms (HR 0.98, 95%CI 0.8–1.2, *p* = 0.84), with freedom from locoregional progression favouring also the surgical treatment arms. Furthermore, a high concurrent risk of distant metastases worsens the cancer specific survival of patients with loco-regionally controlled, resected squamous cell carcinoma [12]. In the present study, the competing risk of distant metastasis was as high as loco-regional progression.

Although loco-regional failure remains a major risk for patients treated with definitive chemoradiation, radiotherapy dose escalation remains controversial.

Up to now no randomized dose-escalation trials on definitive radiochemotherapy could demonstrate a survival benefit for higher total doses than 50.4 Gy using conventional fractionation [9, 11]. On the other hand, older trials using total radiation doses > 60 Gy showed equivalent survival of high dose radiotherapy and concurrent chemotherapy in comparison to tri-modality treatment for responders to induction chemotherapy. The exploratory analysis of the FFCD 9102 trial supported a dose response relation comparing continuous course radiotherapy to 66 Gy at 2 Gy per fraction or split course hypofractionated radiotherapy to 45 Gy at 3 Gy per fraction [25].

In order to limit the potential toxicity of the increased total dose, we have decided to adapt the high-dose PTV to the extension of the residual gross tumor volume after induction-chemotherapy using 18F-FDG-PET/CT plus a margin of 1.0–1.5 cm in axial and 2.0 cm in cranio-caudal direction. Although the value of PET/CT has not been consistently shown in esophageal cancer, several efforts have been made to improve target volume definition using PET/CT [26–29].

Prospective trials investigating high dose radiotherapy have shown that a small target volume is adequate since even after escalated doses local recurrences are the remaining problem and not regional recurrences outside the fields [30].

Other dose escalation trials with lesser follow-up pointed into the same direction that local recurrences remain a major problem even after high-dose radiotherapy [31, 32].

Irradiation techniques have made significant progress during recent years. While the patients in the present study were treated with 3D-conformal radiotherapy, IMRT and VMAT represent current standards of care. Intensity-modulated radiotherapy techniques have fostered the use of integrated-boost irradiation and are under active investigation in prospective dose-escalation trials [33]. The simultaneous integrated boost (SIB) technique has been developed with the aim to increase the dose to macroscopic tumor (GTV) while simultaneously limiting the dose to normal tissues, particularly heart and lungs. Radiobiological modeling suggested a potential gain of tumor control by dose escalation in the GTV [34] and shall be validated in ongoing clinical trials [35].

PET/CT, especially during an induction phase of combined treatment, has been shown to carry prognostic information for definitive and neoadjuvant radiochemotherapy of esophageal squamous cell carcinomas [20, 36, 37]. Our study, although of limited size, confirms that FDG-response to induction treatment is able to separate patient groups with significantly improved prognosis after definitive chemoradiation in relation to patients with non-responding tumors. Prospectively, this might become a valid selection criterion for more or less aggressive local therapy.

## Conclusions

From the present trial, induction chemotherapy using irinotecan (80 mg/m^2^), folinic acid (500 mg/m^2^) and 5-fluorouracil (5-FU, 2 g/m^2^) weekly and cisplatin followed by dose escalated radiotherapy and concomitant cisplatin and irinotecan was tolerable but there was no signal for an improved loco-regional progression-free survival. The benchmark progression-free survival at one year of 74% was not reached. Metabolic response of the tumor after induction chemotherapy was validated as a prognostic factor.

## Data Availability

The datasets generated and/or analysed during the current study are not publicly available because individual privacy could be compromised but are available from the corresponding author on reasonable request.
